# Development of a clinical decision tool to reduce diagnostic testing for primary aldosteronism in patients with difficult-to-control hypertension

**DOI:** 10.1186/s12902-020-0528-3

**Published:** 2020-04-29

**Authors:** Monique E. A. M. van Kleef, Frank L. J. Visseren, Jan Westerink, Michiel L. Bots, Peter J. Blankestijn, Yolanda van der Graaf, Wilko Spiering

**Affiliations:** 1 Department of Vascular Medicine, University Medical Center Utrecht, Utrecht University, 85500, 3508 GA Utrecht, The Netherlands; 2 Julius Center for Health Sciences and Primary Care, University Medical Center Utrecht, Utrecht University, Utrecht, The Netherlands; 3 Department of Nephrology, University Medical Center Utrecht, Utrecht University, Utrecht, The Netherlands

**Keywords:** Primary aldosteronism, Aldosterone-to-renin ratio, Saline infusion test, Difficult-to-control hypertension, Clinical decision tool, Diagnostic test

## Abstract

**Background:**

Satisfactory tools to preclude low-risk patients from intensive diagnostic testing for primary aldosteronism (PA) are lacking. Therefore, we aimed to develop a decision tool to determine which patients with difficult-to-control hypertension have a low probability of PA, thereby limiting the exposure to invasive testing while at the same time increasing the efficiency of testing in the remaining patients.

**Methods:**

Data from consecutive patients with difficult-to-control hypertension, analysed through a standardized diagnostic protocol between January 2010 and October 2017 (*n* = 824), were included in this cross-sectional study. PA was diagnosed by a combined approach: 1) elevated aldosterone-to-renin ratio (> 5.0 pmol/fmol/s), confirmed with 2) non-suppressible aldosterone after standardized saline infusion (≥280 pmol/L). Multivariable logistic regression analyses including seven pre-specified clinical variables (age, systolic blood pressure, serum potassium, potassium supplementation, serum sodium, eGFR and HbA1c) was performed. After correction for optimism, test reliability, discriminative performance and test characteristics were determined.

**Results:**

PA was diagnosed in 40 (4.9%) of 824 patients. Predicted probabilities of PA agreed well with observed frequencies and the c-statistic was 0.77 (95% confidence interval (95%CI) 0.70–0.83). Predicted probability cut-off values of 1.0–2.5% prevented unnecessary testing in 8–32% of the patients with difficult-to-control hypertension, carrying sensitivities of 0.98 (95%CI 0.96–0.99) and 0.92 (0.83–0.97), and negative predictive values of 0.99 (0.98–1.00) and 0.99 (0.97–0.99).

**Conclusions:**

With a decision tool, based on seven easy-to-measure clinical variables, patients with a low probability of PA can be reliably selected and a considerable proportion of patients with difficult-to-control hypertension can be spared intensive diagnostic testing.

## Background

Primary aldosteronism (PA) is one of the most common causes of secondary hypertension. The estimated prevalence ranges from 3–6% in primary care [1, 2] to 5–11% in referred hypertensive patients [3, 4] and is even higher in patients with resistant hypertension (more than 11%) [5]. Diagnosing PA is important as targeted therapy may lower the increased risk for cardiovascular events and target organ damage [6] in these patients.

As supported by the Endocrine Society Clinical Practice Guideline, diagnosis of PA is generally made by a sequence of blood tests: elevated aldosterone-to-renin ratio (ARR) [7], followed by confirmation of autonomous aldosterone production by non-suppressible aldosterone levels [8]. Many antihypertensive medications change renin and aldosterone levels, falsely increasing or decreasing the ARR, which leads to false positive or false negative test results [9, 10]. Therefore, aldosterone and renin levels are usually determined after cessation of antihypertensive medications that affect the ARR [8]. However, medication washout raises safety concerns in patients with severe hypertension or individuals with a recent cardiovascular event in which tight blood pressure (BP) control is necessary. In addition, a substantial number of patients experience symptoms during the washout period: i.e. (worsening of) headache, fatigue or palpitations.

Since diagnostic testing is burdensome, potentially harmful and costly, it is necessary to select carefully which patients to further test for PA. The Endocrine Society Clinical Practice Guideline recommends to perform diagnostic testing in all patients with ‘an increased risk of having PA’, this includes patients with hypertension and either sustained BP above 150/100 mmHg, spontaneous or diuretic-induced hypokalemia, adrenal incidentaloma, obstructive sleep apnea or a family history of early onset hypertension or cerebrovascular accident, patients with resistant hypertension and first-degree relatives of patients with PA [8]. The majority of hypertensive patients referred to hospitals fulfil at least one of these criteria, meaning that most referred patients should be tested for PA. With a prevalence of 4–10% this strategy results in many negative test results and thus to unnecessary testing and costs. This may be one of the reasons that the Clinical Practice Guideline is poorly adopted and that many patients with hypertension are left unscreened for PA [11]. Therefore, we aimed to develop and validate a clinical decision tool to determine which patients with difficult-to-control hypertension have a low probability of PA and do not need to undergo intensive testing. Thereby we aim to limit exposure to invasive testing while at the same time increasing the efficiency of testing in the remaining patients.

## Methods

### Study population

1125 hypertensive patients were referred to the University Medical Center Utrecht between January 2010 and October 2017. Patients with difficult-to-control hypertension, defined as persistent hypertension despite treatment according to the current guidelines and/or presence of (sub) clinical vascular disease [12], were eligible for this cross-sectional study. These patients reflect the group of patients commonly referred by general practitioners. Patients with difficult-to-control hypertension underwent an extensive, standardized diagnostic protocol to evaluate the cause of hypertension. The diagnostic protocol has been outlined in detail previously [13]. In general, patient selection for this protocol was in line with the Endocrine Society Clinical Practice Guideline recommendations, which recommends widespread screening for PA. Since participants in this study were not subject to procedures and were not required to follow rules of behaviour outside the scope of routine clinical practice, no formal consent was required [14, 15], which was approved by the institutional ethics committee (Medisch Ethische Toetsingscommissie Utrecht, University Medical Center Utrecht, Utrecht, The Netherlands). Patients who were normotensive on 24-h ambulatory BP measurement, patients with an evident and treatable cause of hypertension such as steroid or excessive liquorice use, and patients with a recent cardiovascular event in which withdrawal of antihypertensive medication was not safe, did not undergo the extensive, standardized diagnostic work-up and were excluded from this study (*n* = 299).

### Baseline measurements

During the first hospital visit information on patient demographics, medical history and medication use was collected. Office BP was measured with an automated oscillometric device (Omron M7 Intelli IT, OMRON Healthcare, Hoofddorp, Netherlands; or WatchBP Office, Microlife, Widnau, Switzerland) after five min rest while the patient was seated. Three readings were recorded, simultaneously on both arms, and one min apart. The third BP measurement on the arm that measured the highest BP was recorded. 24-h ambulatory BP measurement (WatchBP O3 Ambulatory, Microlife, Widnau, Switzerland) was also performed on the arm that measured the highest BP and recorded BP every 20 to 30 min, day and night. Screening for obstructive sleep apnea was performed with the Philips questionnaire. Patients at intermediate or high risk for obstructive sleep apnea as determined by this questionnaire, were additionally screened by the overnight RUSleeping RTS [16]. Patients with more than 15 events per hour were considered having probable obstructive sleep apnea. Blood tests were performed and included fasting glucose, HbA1c, sodium, potassium, creatinine, cholesterol levels, TSH and fT4. Urinary sodium, albumin and creatinine were determined in the first morning-void urine sample. Calculated albumin-to-creatinine ratios were categorized into category 1 (< 3 mg/mmol), category 2 (3–30 mg/mmol) or category 3 (≥30 mg/mmol).

The diagnostic protocol was changed in June 2015. Before that time, the protocolised set of laboratory tests was performed during the medication washout period. Baseline laboratory tests and pre-washout 24-h ambulatory BP measurements were only performed as indicated.

### Diagnosis of primary aldosteronism

Aldosterone and renin levels were measured after cessation of antihypertensive medication for 2 to 6 weeks: 2 weeks for ACE inhibitors, angiotensin II antagonists, calcium channel blockers, alpha blockers and direct vasodilators; 4 weeks for diuretics and beta blockers (the latter including a two-week tapering scheme); and 6 weeks for mineralocorticoid receptor antagonists and direct renin inhibitors. In addition, oral contraceptives and NSAIDs were discontinued for at least six respectively 2 weeks. If BP rose above the predetermined BP level or if the patient experienced hypertensive symptoms, diltiazem and/or doxazosin [9] were prescribed. Plasma aldosterone concentration (PAC; pmol/L) and plasma renin activity (PRA; fmol/L/s) were measured seated in the early morning after patients had been up for at least 90 min and after correction of hypokalemia (potassium < 3.8 mmol/L). PAC and PRA were both measured using a radioimmunoassay, the methodology has been described elsewhere [17]. The ARR was calculated by dividing PAC by PRA. The ARR cut-off value was 5 pmol/fmol/s, which has been shown to reach a sensitivity of 100% in our laboratory [17]. The diagnosis of PA was confirmed by a non-suppressible aldosterone (≥280 pmol/L) after salt loading (SLT) with two liters intravenous saline (0.9%) infusion in 4 h [8]. A CT-scan with/without adrenal venous sampling (AVS) was performed to distinguish between unilateral PA (i.e. aldosterone-producing adenomas or unilateral adrenal hyperplasia) and bilateral PA (bilateral adrenal hyperplasia).

### Statistical analyses

The decision tool was built with the following pre-specified clinical variables: age, 24-h ambulatory systolic BP, serum potassium, potassium supplementation (yes/no), serum sodium, eGFR and HbA1c. These variables were chosen based on findings from previous studies in which they significantly differed between patients with PA and primary hypertension and/or were independently associated with PA [3, 18–20]. More importantly, these clinical variables are easy to obtain in the hospital setting as well as in general practice, enabling widespread use of the diagnostic model.

Patients with a missing reference test (ARR or saline infusion test result after elevated ARR) were excluded from analysis (*n* = 2). Baseline characteristics are given for the observed, non-imputed data. Missing values for the variables in the model were assumed to be missing at random conditional on other observed variables and/or the outcome. For further analysis, missing values (age, *n* = 1; 24-h ambulatory systolic BP, *n* = 497; serum potassium, *n* = 451; potassium supplementation, *n* = 0; serum sodium, *n* = 454; eGFR, *n* = 413; and HbA1c, *n* = 76) were imputed using 20-fold multiple imputation by predictive mean matching (for continuous variables) and polytomous regression (for categorical variables) (R-package MICE). A complete case analysis excluding these patients would yield loss of efficiency and would provide biased results, since missing data rarely occur completely at random and are usually dependent on the outcome [21]. The imputation model included the seven clinical characteristics for the decision tool, the outcome, and several other clinical variables collected during the outpatient visits [22] including prescribed antihypertensive medication, measures of target organ damage and laboratory values measured during medication washout. Ambulatory blood pressure and laboratory values of potassium, sodium and creatinine measured during medication washout were available for > 96 and > 99% of the patients with missing baseline values. Primarily using these follow-up values to build the decision tool would provide incorrect estimates as medication withdrawal changes the laboratory values.

Model derivation was performed by multivariable logistic regression, including the seven pre-specified clinical variables. A separate model was fit on each multiply imputed dataset. No variable selection was performed. Variables were logarithmically or quadratically transformed if this improved overall model fit, determined by Akaike’s Information Criterion. Internal validation was performed by applying a bootstrap-based shrinkage technique [23, 24]. The intercept was adjusted after recalibration.

Model performance was assessed by discrimination and calibration. In addition, test characteristics for different cut-off values of the predicted probability were determined. Discriminative performance was estimated by pooling the c-statistics from each dataset using Rubin’s rule. Test characteristics (positive and negative predictive value (PPV and NPV), positive and negative likelihood ratio (LR+ and LR-), sensitivity and specificity) were obtained similarly. These estimates and their standard errors (except LR+) were logit transformed, pooled by using Rubin’s rule, and then back transformed [25]. The calibration plot was obtained by plotting the observed frequencies of PA against the pooled predictions of the 20 imputed datasets. The final model was presented after pooling the shrunken beta coefficients, recalibrated intercepts and standard errors through Rubin’s rule.

Since other centers may use different post-SLT aldosterone cut-off values to increase sensitivity of the confirmation test, sensitivity analysis was performed by using the final model to predict PA defined as ARR > 5 pmol/fmol/sec confirmed by post-SLT aldosterone ≥190 pmol/L. Predicted probabilities, c-statistic and test characteristics were obtained by applying the final model to the 20 imputed datasets and pooling the results in a similar way as described above. All statistical analyses were performed with R, version 3.4.3 (R Development Core Team, Vienna, Austria).

## Results

### Baseline characteristics and prevalence of PA

Baseline characteristics for patients included in this cross-sectional, diagnostic study differed from those who were excluded: age 53.2 (±13.3) vs 58.1 (±15.9) years, family history of hypertension 66% vs 54%, 24-h ambulatory BP 144/86 (±17/11) vs 134/78 (±18/10) mmHg and probable obstructive sleep apnea 19% vs 42% (Supplementary File 1). Although the study population comprised 49% women, the proportion of women among the PA cases was only 20% (Table 1). The majority of the patients (94% in the original, non-imputed dataset) fulfilled the Endocrine Society Guidelines of increased risk for PA. 137 of 824 patients (17%) had an elevated ARR (> 5 pmol/fmol/s) and 40 patients (4.9%) had a confirmed diagnosis of PA after saline infusion. AVS was performed in 29 patients, 17 showed lateralization and were therefore diagnosed as having unilateral PA. In 10 patients PA subtyping was based on the CT-scan, which showed a unilaterally enlarged adrenal gland in four patients. One patient did not undergo additional subtyping.

**Table 1:** Patient characteristics summarized for the total population and patients with and without primary aldosteronism

	Total population	Primary aldosteronism	No primary aldosteronism
	**(*n*** **= 824)**	**(*n*** **= 40)**	**(*n*** **= 784)**
Age (years)	53.2 (13.3)	53.9 (10.1)	53.2 (13.5)
Sex (female)	405 (49%)	8 (20%)	397 (51%)
Family history of hypertension	510 (66%)	27 (69%)	483 (66%)
Office blood pressure (mmHg)	171/98 (26/14)	168/103 (21/13)	171/98 (26/14)
Office heart rate (bpm)	74 (14)	68 (10)	74 (14)
24-h ambulatory blood pressure (mmHg)	144/86 (17/11)	149/90 (16/8)	144/86 (17/12)
Dipping (%)^a^	11 (7)	10 (7)	11 (7)
Number of antihypertensive medication classes^b^	2 (1–3)	2 (1–3)	2 (1–3)
ACE-inhibitor / ARB / direct renin inhibitor	572 (69%)	27 (68%)	545 (70%)
Diuretic	385 (47%)	18 (45%)	367 (47%)
Potassium-sparing diuretic	22 (3%)	0 (0%)	22 (3%)
Mineralocorticoid antagonist	93 (11%)	6 (15%)	87 (11%)
BMI (kg/m^2^)	28 (5)	30 (4)	28 (5)
HbA1c (mmol/mol)	37 (33–40)	36 (33–39)	37 (33–40)
Probable obstructive sleep apnea^c^	125 (19%)	11 (34%)	114 (19%)
Serum sodium (mmol/L)	139 (3)	140 (2)	139 (3)
Serum potassium (mmol/L)	3.9 (0.4)	3.5 (0.5)	4.0 (0.4)
Hypokalemia (< 3.5 mmol/L)	40 (11%)	9 (45%)	31 (9%)
Potassium supplementation	9 (1%)	3 (8%)	6 (1%)
eGFR (mmol/L/1.73m^2^)^d^	84 (20)	83 (20)	84 (20)
Albuminuria category 2 (ACR 3–30 mg/mmol)	117 (19%)	10 (30%)	107 (19%)
Albuminuria category 3 (ACR > 30 mg/mmol)	25 (4%)	3 (9%)	22 (4%)
Use of escape medication	176 (22%)	14 (36%)	162 (21%)
Aldosterone/renin ratio > 5 pmol/fmol/s	137 (17%)	40 (100%)	97 (12%)
Plasma aldosterone after salt loading test (pmol/L)	170 (90–290)	365 (310–635)	110 (70–180)
Fulfill Endocrine Society Guideline Criteria	687 (94%)	34 (100%)	653 (93%)

Based on non-imputed data, data are presented as mean ± SD, median (IQR) or n (%) for the patients with non-missing values for that characteristic. ^a^Dipping defined as mean BP at daytime minus mean BP at night-time divided by mean BP at daytime * 100%. ^b^Antihypertensive medication classes divided into: ACE-inhibitors or angiotensin receptor blockers, calcium channel blockers, diuretics, mineralocorticoid receptor antagonists, beta blockers, alpha blockers, direct renin inhibitors, direct vasodilators, or central acting antihypertensive drugs. ^c^Intermediate to high risk determined by the Philips questionnaire and RUSleeping RTS showing > 15 apneas per hour. ^d^Estimated glomerular filtration rate by Chronic Kidney Disease Epidemiology Collaboration equation. ACR = albumin-to-creatinine ratio.

### Development and validation of the diagnostic model

The average shrinkage factor over the 20 imputed datasets was 0.78, resulting in the following diagnostic model after pooling:

Predicted probability = 1/(1 + exp-(− 27.3348 + 0.2082*age (in years) – 0.0021*age (in years)^*2*^ + 0.0138*24-h ambulatory systolic BP (in mmHg) – 0.8296*potassium (in mmol/L) + 1.5057 (if potassium is supplemented) + 0.1593*sodium (in mmol/L) – 0.0103*eGFR (in mmol/L) – 0.0246*HbA1c (in mmol/mol))).

**Fig. 1:**
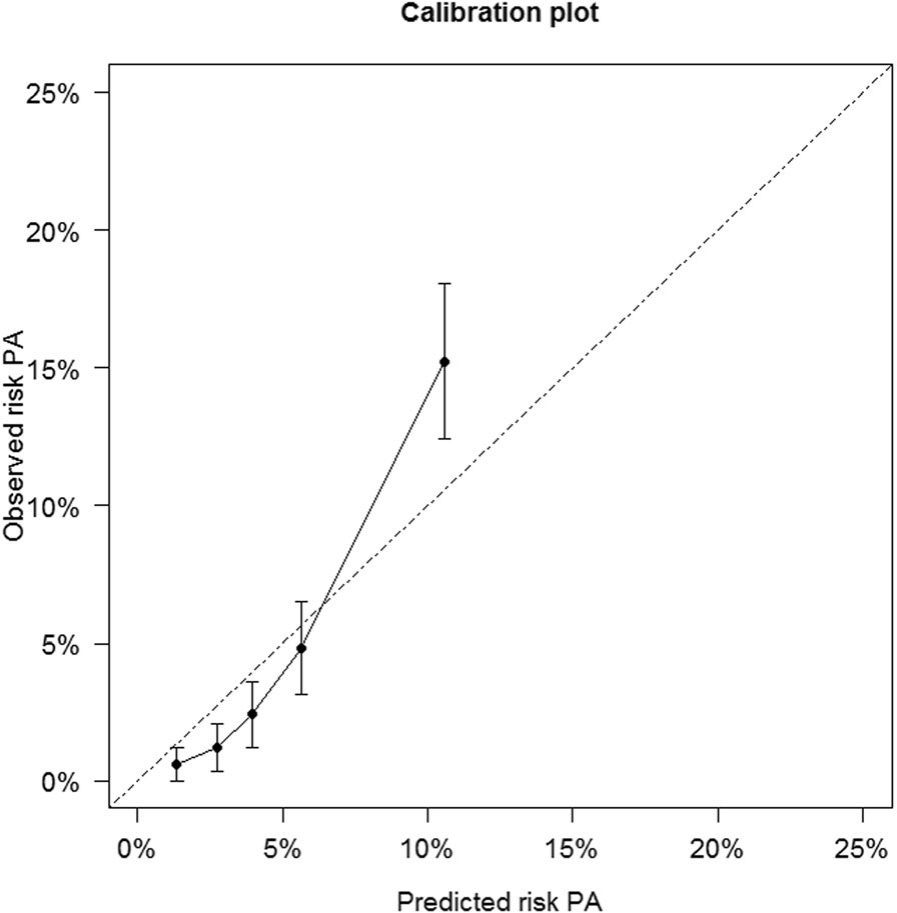
Calibration plot showing the agreement between predicted and observed probabilities of primary aldosteronism. Error bars represent corresponding Bootstrap-based standard errors. PA = primary aldosteronism

**Fig. 2:**
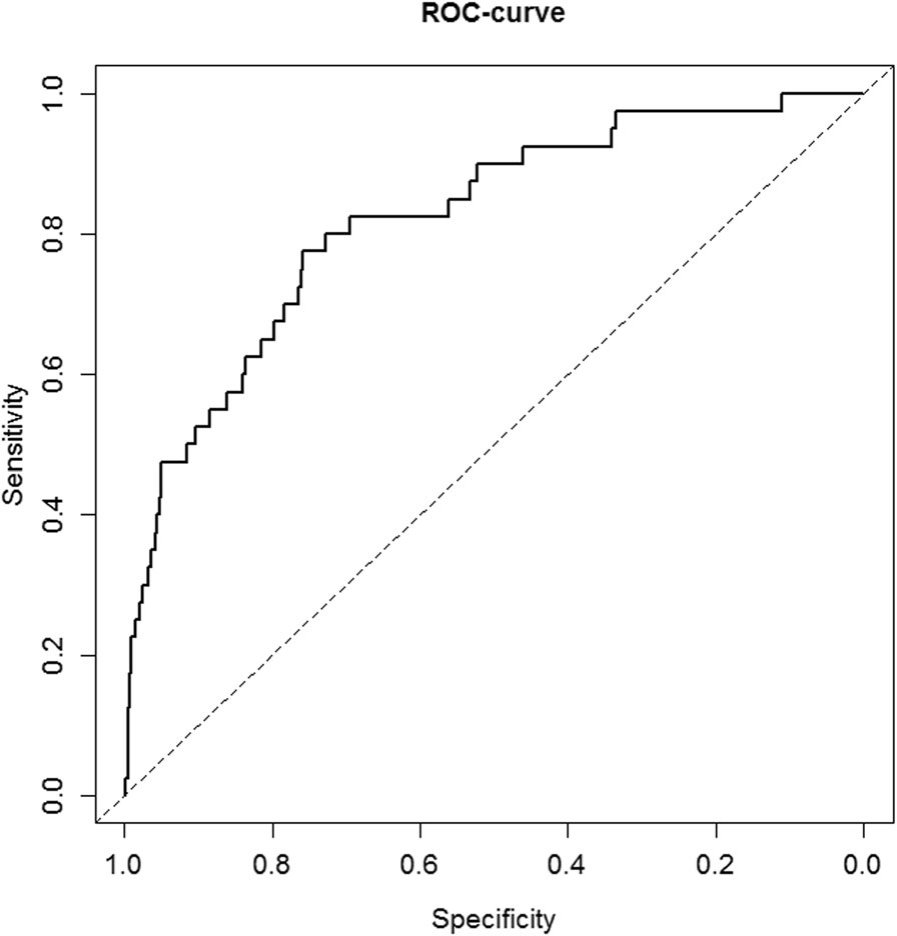
Receiver operating characteristics (ROC) curve showing the discriminative performance of the diagnostic tool. Discriminative performance is the ability of the model to distinguish between patients with and without primary aldosteronism. The ROC curve plots the sensitivity vs specificity for different cut-off values of the tool (predicted probabilities)

The predicted probability for an individual patient can be calculated by using the calculator provided in Supplementary File 2. Pooled model coefficients and odds ratios are presented in Table 2. The calibration plot (Fig. 1) shows the agreement between predicted probabilities and observed frequencies of PA*.* The discriminative ability of the diagnostic tool was moderate to good with a c-statistic of 0.77 (95%CI 0.70–0.83) (Fig. 2). Table 3 shows the test characteristics (sensitivity, specificity, PPV, NPV, LR+ and LR-) and proportion of patients spared intensive testing for predicted probability cut-off values between 1.0 and 2.5%. This range is chosen as this is the zone where clinical decision making is going on. The proportion of patients spared intensive testing reflects the proportion of patients with a predicted probability equal to or below the cut-off value in which (according to our decision tool) no further testing is needed, and is estimated at 8% (95%CI 4–18%) to 32% (18–50%). These cut-off values carry a sensitivity of 0.98 (95%CI 0.96–0.99) and 0.92 (0.83–0.97), and NPV of 0.99 (0.98–1.00) and 0.99 (0.97–0.99). Sensitivity analysis predicting PA when a lower post-SLT aldosterone cut-off value (≥190 pmol/L) was applied, hardly changed sensitivity and NPV, and demonstrated similar agreement (Supplementary files 3–4).

**Table 2:** Model coefficients and odds ratios

	coefficient	odds ratio (95% CI)
Intercept	−27.3348	
Age (per year)	0.2082	
Age^2^ (per year)^2^	−0.0021	
24-h ambulatory systolic BP (per mmHg)	0.0138	1.01 (0.99–1.04)
Potassium (per 0.1 mmol/L)	−0.0830	0.92 (0.83–1.02)
Potassium supplementation (yes)	1.5057	4.51 (0.94–21.68)
Sodium (per mmol/L)	0.1593	1.17 (0.98–1.41)
eGFR (per 10 ml/min/1.73 m^2^)^a^	− 0.1027	0.90 (0.70–1.16)
HbA1c (per mmol/mol)	−0.0246	0.98 (0.92–1.04)

Pooled beta coefficients and odds ratios (OR) for the different clinical characteristics in the shrunken multivariate logistic regression model. ^a^Estimated glomerular filtration rate by Chronic Kidney Disease Epidemiology Collaboration eq. BP Blood pressure.

**Table 3:** Test characteristics and proportion of patients spared intensive testing

	Cut-off value of the predicted probability			
	1.0%	1.5%	2.0%	2.5%
**Sensitivity**	0.98 (0.96–0.99)	0.97 (0.91–0.99)	0.95 (0.89–0.98)	0.92 (0.83–0.97)
**Specificity**	0.09 (0.04–0.19)	0.16 (0.07–0.32)	0.25 (0.13–0.43)	0.33 (0.19–0.52)
**Positive predictive value**	0.05 (0.05–0.06)	0.06 (0.05–0.06)	0.06 (0.05–0.07)	0.07 (0.05–0.08)
**Negative predictive value**	0.99 (0.98–1.00)	0.99 (0.98–1.00)	0.99 (0.98–1.00)	0.99 (0.97–0.99)
**Positive likelihood ratio**	1.08 (1.00–1.17)	1.17 (1.01–1.33)	1.28 (1.04–1.52)	1.40 (1.06–1.75)
**Negative likelihood ratio**	0.18 (0.07–0.39)	0.19 (0.08–0.38)	0.20 (0.08–0.43)	0.24 (0.09–0.49)
**Proportion of patients spared intensive testing**	8% (4–18)	15% (7–31)	24% (12–41)	32% (18–50)

The positive likelihood ratio tells you how much to increase the probability of having a disease, given a positive test result. The negative likelihood ratio tells you how much to decrease the probability of having a disease, given a negative test result. The proportion of patients spared intensive testing is the proportion of patients with a predicted probability equal to or below the cut-off value. Estimates and corresponding Bootstrap-based 95% confidence intervals are presented for different cut-off values of the predicted probability by the diagnostic tool.

## Discussion

The main finding of this cross-sectional, diagnostic study is that a decision tool with seven easy-to-measure clinical variables can reliably select patients with difficult-to-control hypertension with a low probability of PA, sparing 8 to 32% of patients intensive diagnostic testing.

This is an important finding since satisfactory tools to preclude low-risk patients from diagnostic testing are lacking. There is a need for diagnostic tools that reduce the number of patients to be intensively tested for PA to a greater extent than the algorithm provided by the Endocrine Society does. Moreover, results from a French study deriving a diagnostic model to estimate the probability of PA in patients referred for PA screening [19], cannot be generalized to all patients referred with difficult-to-control hypertension. The investigators included a study population with a substantial higher prevalence of PA (elevated ARR was 45% compared to 17% in our study) and a relatively large proportion of patients on potassium supplementation. Moreover, they predicted the presence of elevated ARR instead of PA itself.

The population of patients with difficult-to-control hypertension in the present study represents the group of hypertensive patients generally referred by general practitioners, which is reflected by the prevalence of PA: 4.9% in this study compared to 4.6–11.2% in other cohorts of referred hypertensive patients [3, 4]. More importantly, the majority of the patients in this study fulfil the Endocrine Society Clinical Practice Guidelines of increased risk for PA [8]. Therefore, the decision tool in the present study adds diagnostic value on top of the diagnostic algorithm presented in the Guideline. The proportion of male PA patients in this study is relatively high, given the small number of PA cases in the present study, the observed difference may also be due to chance.

The clinical value of the decision tool lies in excluding PA, precluding low-risk patients from intensive diagnostic testing, rather than confirming PA. The extent to which it reduces the proportion of patients to be tested varies with the cut-off value chosen and depends on the clinical setting in which the decision tool will be applied. In community hospitals, where the prevalence of PA is relatively low, a high cut-off value (for example 2.5%) may be chosen, sparing intensive diagnostic testing in a considerable proportion of patients, while the absolute number of missed cases remains low. In a tertiary center, where the prevalence of PA is relatively high, a lower cut-off value (for example 1.5%) may be chosen, limiting the chance of a missed case but still reducing the diagnostic burden. By reducing the proportion of hypertensive patients to be intensively tested and increasing the probability of catching a PA case among those tested, our decision tool may motivate physicians to perform diagnostic workup for PA in patients with difficult-to-control hypertension, thereby reducing underdiagnoses.

One of the strengths of this study is that the reference test was missing in only two of the 826 patients. Additionally, the chance of missing a PA case based on a false negative ARR is low: sensitivity of the ARR (cut-off value 5 pmol/fmol/s) reached 100% (95%CI 75.9–100%) in our laboratory [17] and hypokalemia was corrected before it was determined. Since all patients were diagnosed by the same combined approach, our results do not suffer from differential verification bias. Yet, the aldosterone cut-off value of ≥280 pmol/L after saline infusion (as proposed by the Endocrine Society Clinical Practice Guideline [8]) on which PA diagnosis was based, is rather arbitrary and results in a small number of false negative test results [26, 27]. Some patients with an intermediate post-SLT aldosterone (140–280 pmol/L) do have the clinical syndrome of PA based on adrenal computed tomography, adrenal venous sampling and/or expert panel assessment [26]. However, sensitivity analysis showed that our decision tool also reliably selects patients at low risk for PA when a lower post-SLT cut-off value is used. In addition, patients with an intermediate test result are more likely to have idiopathic aldosteronism [27], limiting the chance of withholding a surgical cure for these patients, but possibly precluding them from receiving adequate medical treatment with a mineralocorticoid receptor antagonist. Other patients may have pre-stages of PA, illustrating the importance of repeated screening by the decision tool after several months or years. Another strength of this study is that clinical variables were routinely collected, resembling daily clinical practice, which is critical in a diagnostic study.

One of the limitations of this study, as in any study using multiple imputation methods to impute missing baseline values, is that the robustness of the method highly depends on the validity of the missing at random assumption. Although our diagnostic protocol was changed in June 2015 and many patients had missing values before that time, one third of the patients evaluated before June 2015 did have a complete set of baseline values. Therefore, these missings can be considered missing at random. Furthermore, although pre-specified clinical variables were used to minimize the risk of overfitting, the methodology by which these variables were selected was not systematic and is therefore vulnerable to bias. The lack of statistical significance for some of the pre-specified variables in the model can be the result of this unsystematic selection but can also be due to a lack of power. Finally, the model was not externally validated in another cohort of patients with difficult-to-control hypertension, which is required to guarantee generalizability and should be done before the decision tool is applied in clinical practice.

## Conclusions

In conclusion, with a decision tool based on seven easy-to-measure clinical variables, patients with a low probability of PA can be reliably selected and a considerable proportion of patients with difficult-to-control hypertension can be spared further intensive diagnostic testing.

### Future perspectives

By excluding patients at low risk for PA, the decision tool reduces the number of patients to be invasively tested and increases the efficiency of testing in the remaining patients, which will allow health-care providers to allocate their (financial) resources to patients with difficult-to-control hypertension at higher risk for PA. This work raises the opportunity for external validation in other patient populations (i.e. primary care) and may lead to extensive clinical application, better detection and, subsequently, earlier treatment of PA. Also, given the high prevalence of PA in drug-adherent patients as compared to non-adherent patients [28], adding a measure of adherence to the existing model may further improve the diagnostic accuracy of the decision tool. That may decrease the number of patients that need invasive testing even more.

## Supplementary information

**Additional file 1.** Non-imputed patient characteristics summarized for the patients included in and excluded from this study.

**Additional file 2.** Primary aldosteronism probability calculator. Excel sheet for calculating the predicted probability of primary aldosteronism for an individual patient with difficult-to-control hypertension.

**Additional file 3.** Test characteristics and proportion of patients spared intensive testing resulting from sensitivity analysis.

**Additional file 4.** Calibration plot showing the agreement after sensitivity analysis. It shows the agreement between predicted and observed probabilities of primary aldosteronism when a post-salt loading test aldosterone cut-off value of ≥190 pmol/L is applied. The error bars represent the corresponding Bootstrap-based standard errors. PA = primary aldosteronism.

## Data Availability

The datasets used and/or analysed during the current study are available from the corresponding author on reasonable request.

## Abbreviations

ARR: Aldosterone-to-renin ratio
AVS: Adrenal venous sampling
BP: Blood pressure
NPV: Negative predictive value
PA: Primary aldosteronism
PAC: Plasma aldosterone concentration
PPV: Positive predictive value
PRA: Plasma renin activity
SLT: Salt loading test

## References

[1] Fogari R, Preti P, Zoppi A, Rinaldi A, Fogari E, Mugellini A (2007). Prevalence of primary aldosteronism among unselected hypertensive patients: a prospective study based on the use of an aldosterone/renin ratio above 25 as a screening test. Hypertens Res.

[2] Kayser SC, Deinum J, de Grauw WJ, Schalk BW, Bor HJ, Lenders JW (2018). Prevalence of primary aldosteronism in primary care: a cross-sectional study. Br J Gen Pract.

[3] Rossi GP, Bernini G, Caliumi C, Desideri G, Fabris B, Ferri C (2006). A prospective study of the prevalence of primary aldosteronism in 1,125 hypertensive patients. J Am Coll Cardiol.

[4] Mulatero P, Stowasser M, Loh KC, Fardella CE, Gordon RD, Mosso L (2004). Increased diagnosis of primary aldosteronism, including surgically correctable forms, in centers from five continents. J Clin Endocrinol Metab.

[5] Douma S, Petidis K, Doumas M, Papaefthimiou P, Triantafyllou A, Kartali N (2008). Prevalence of primary hyperaldosteronism in resistant hypertension: a retrospective observational study. Lancet..

[6] Monticone S, D'Ascenzo F, Moretti C, Williams TA, Veglio F, Gaita F (2018). Cardiovascular events and target organ damage in primary aldosteronism compared with essential hypertension: a systematic review and meta-analysis. Lancet Diabetes Endocrinol.

[7] Hiramatsu K, Yamada T, Yukimura Y, Komiya I, Ichikawa K, Ishihara M (1981). A screening test to identify aldosterone-producing adenoma by measuring plasma renin activity. Results in hypertensive patients. Arch Intern Med.

[8] Funder JW, Carey RM, Mantero F, Murad MH, Reincke M, Shibata H (2016). The Management of Primary Aldosteronism: case detection, diagnosis, and treatment: an Endocrine Society clinical practice guideline. J Clin Endocrinol Metab.

[9] Mulatero P, Rabbia F, Milan A, Paglieri C, Morello F, Chiandussi L (2002). Drug effects on aldosterone/plasma renin activity ratio in primary aldosteronism. Hypertension..

[10] Jansen PM, van den Born BJ, Frenkel WJ, de Bruijne EL, Deinum J, Kerstens MN (2014). Test characteristics of the aldosterone-to-renin ratio as a screening test for primary aldosteronism. J Hypertens.

[11] Mulatero P, Monticone S, Burrello J, Veglio F, Williams TA, Funder J (2016). Guidelines for primary aldosteronism: uptake by primary care physicians in Europe. J Hypertens.

[12] Beeftink MM, van der Sande NG, Bots ML, Doevendans PA, Blankestijn PJ, Visseren FL (2017). Safety of temporary discontinuation of antihypertensive medication in patients with difficult-to-control hypertension. Hypertension..

[13] Van Der Sande NGC, Blankestijn PJ, Visseren FLJ, Beeftink MM, Voskuil M, Westerink J (2019). Prevalence of potential modifiable factors of hypertension in patients with difficult-to-control hypertension. J Hypertens.

[14] Subjects CCoRIH. Legal framework for medical scientific research: Your research: Is it subject to the WMO or not? [Available from: https://english.ccmo.nl/investigators/legal-framework-for-medical-scientific-research/your-research-is-it-subject-to-the-wmo-or-not.

[15] Overheid.nl. Wettenbank - Wet medisch-wetenschappelijk onderzoek met mensen 2020 [updated January 1, 2020. Available from: https://wetten.overheid.nl/BWBR0009408/2020-01-01.

[16] Grover SS, Pittman SD (2008). Automated detection of sleep disordered breathing using a nasal pressure monitoring device. Sleep Breath.

[17] Vorselaars W, Valk GD, Vriens MR, Westerink J, Spiering W (2018). Case detection in primary aldosteronism: high-diagnostic value of the aldosterone-to-renin ratio when performed under standardized conditions. J Hypertens.

[18] Hanslik G, Wallaschofski H, Dietz A, Riester A, Reincke M, Allolio B (2015). Increased prevalence of diabetes mellitus and the metabolic syndrome in patients with primary aldosteronism of the German Conn's registry. Eur J Endocrinol.

[19] Ducher M, Mounier-Vehier C, Lantelme P, Vaisse B, Baguet JP, Fauvel JP (2015). Reliability of a Bayesian network to predict an elevated aldosterone-to-renin ratio. Arch Cardiovasc Dis.

[20] Rossi GP, Bernini G, Desideri G, Fabris B, Ferri C, Giacchetti G (2006). Renal damage in primary aldosteronism: results of the PAPY study. Hypertension..

[21] Donders AR, van der Heijden GJ, Stijnen T, Moons KG (2006). Review: a gentle introduction to imputation of missing values. J Clin Epidemiol.

[22] van Buuren S (2007). Multiple imputation of discrete and continuous data by fully conditional specification. Stat Methods Med Res.

[23] Steyerberg EW, Eijkemans MJ, Harrell FE, Habbema JD (2001). Prognostic modeling with logistic regression analysis: in search of a sensible strategy in small data sets. Med Decis Mak.

[24] Schomaker M, Heumann C (2018). Bootstrap inference when using multiple imputation. Stat Med.

[25] Wood AM, Royston P, White IR (2015). The estimation and use of predictions for the assessment of model performance using large samples with multiply imputed data. Biom J.

[26] Giacchetti G, Ronconi V, Lucarelli G, Boscaro M, Mantero F (2006). Analysis of screening and confirmatory tests in the diagnosis of primary aldosteronism: need for a standardized protocol. J Hypertens.

[27] Rossi GR, Bernini GP, Caliumi C, Desideri GB, Fabris B, Giacchetti G (2006). Prospective assessment of the diagnostic performance of the saline infusion test in the primary aldosteronism prevalence in hypertensives (PAPY) study. J Hypertens.

[28] Velasco A, Chung O, Raza F, Pandey A, Brinker S, Arbique D (2015). Cost-effectiveness of therapeutic drug monitoring in diagnosing primary Aldosteronism in patients with resistant hypertension. J Clin Hypertens.

