# Supplementary material for: Development of a clinical decision tool to reduce diagnostic testing for primary aldosteronism in patients with difficult-to-control hypertension
Source: BMC Endocr Disord. 2020 Apr 29;20:56. doi: 10.1186/s12902-020-0528-3 (PMC7191700; doi:10.1186/s12902-020-0528-3)
Supplement: Supplementary file 1 — Additional file 1. Non-imputed patient characteristics summarized for the patients included in and excluded from this study. [file 12902_2020_528_MOESM1_ESM.docx]

**Supplementary File 1** - **Non-imputed patient characteristics summarized for the patients included in and excluded from this study.**

|  | **Study population** | **Mis-sings (%)** | **Excluded patients** | **Mis-sings (%)** |
| --- | --- | --- | --- | --- |
|  |  |  |  |  |
|  | **(n = 824)** |  | **(n = 301)** |  |
| Age (years) | 53.2 (13.3) | 0 | 58.1 (15.9) | 15 |
| Sex (female) | 405 (49%) | 0 | 166 (55%) | 0 |
| Family history of hypertension | 510 (66%) | 6 | 141 (54%) | 13 |
| Office blood pressure (mmHg) | 171/98 (26/14) | 5 | 161/92 (28/14) | 34 |
| Office heart rate (bpm) | 74 (14) | 14 | 72 (13) | 36 |
| 24-hour ambulatory blood pressure (mmHg) | 144/86 (17/11) | 60 | 134/78 (18/10) | 41 |
| Dipping (%)^a^ | 11 (7) | 60 | 11 (9) | 41 |
| Number of antihypertensive medication classes^b^ | 2 (1 - 3) | 0 | 2 (0 - 3) | 48 |
| ACE-inhibitor / ARB / direct renin inhibitor | 573 (70%) | 0 | 83 (53%) | 48 |
| Diuretic | 385 (47%) | 0 | 62 (40%) | 48 |
| Potassium-sparing diuretic | 22 (3%) | 0 | 4 (3%) | 48 |
| Mineralocorticoid antagonist | 93 (11%) | 0 | 17 (11%) | 48 |
| BMI (kg/m^2^) | 28 (5) | 1 | 28 (5) | 19 |
| HbA1c (mmol/mol) | 37 (33 - 40) | 9 | 34 (31 - 35) | 95 |
| Probable obstructive sleep apnea^c^ | 125 (19%) | 22 | 84 (42%) | 33 |
| Serum sodium (mmol/L) | 139 (3) | 55 | 138 (3) | 23 |
| Serum potassium (mmol/L) | 3.9 (0.4) | 55 | 4.0 (0.4) | 21 |
| Hypokalemia (< 3.5 mmol/L) | 40 (11%) | 55 | 11 (5%) | 21 |
| Potassium supplementation | 9 (1%) | 0 | 5 (2%) | 0 |
| eGFR (mmol/L/1.73m^2^)^d^ | 84 (20) | 50 | 79 (23) | 24 |
| Albuminuria category 2 (ACR 3-30 mg/mmol) | 117 (19%) | 27 | 3 (27%) | 96 |
| Albuminuria category 3 (ACR >30 mg/mmol) | 25 (4%) | 27 | 0 (0%) | 96 |
| Use of escape medication | 176 (22%) | 3 | NA | NA |
| Aldosterone/renin ratio >5 pmol/fmol/s | 137 (17%) | 0 | NA | NA |
| Plasma aldosterone after salt loading test (pmol/L) | 170 (90 - 290) | 83 | NA | NA |
| Fulfill Endocrine Society Guideline Criteria | 687 (94%) | 11 | 155 (90%) | 43 |

*Data are presented as mean ± SD, median (IQR) or n (%) for the patients with non-missing values for that characteristic. For each characteristic the proportion of missing values is given (% of total).*

*^a^Dipping defined as mean BP at daytime minus mean BP at night-time divided by mean BP at daytime * 100%.*

*^b^Antihypertensive medication classes divided into: ACE-inhibitors or angiotensin receptor blockers, calcium channel blockers, diuretics, mineralocorticoid receptor antagonists, beta blockers, alpha blockers, direct renin inhibitors, direct vasodilators, or central acting antihypertensive drugs.*

*^c^Intermediate to high risk determined by the Philips questionnaire and RUSleeping RTS showing >15 apneas per hour.*

*^d^Estimated glomerular filtration rate by Chronic Kidney Disease Epidemiology Collaboration equation. ACR = albumin-to-creatinine ratio, NA = not applicable.*
