# Supplementary material for: Development of a clinical decision tool to reduce diagnostic testing for primary aldosteronism in patients with difficult-to-control hypertension
Source: BMC Endocr Disord. 2020 Apr 29;20:56. doi: 10.1186/s12902-020-0528-3 (PMC7191700; doi:10.1186/s12902-020-0528-3)
Supplement: Supplementary file 3 — Additional file 3. Test characteristics and proportion of patients spared intensive testing resulting from sensitivity analysis. [file 12902_2020_528_MOESM3_ESM.docx]

**Supplementary File 3 - Test characteristics and proportion of patients spared intensive testing resulting from sensitivity analysis.**

|  | **Cut-off value of the predicted probability** | | | |
| --- | --- | --- | --- | --- |
|  | **1.0%** | **1.5%** | **2.0%** | **2.5%** |
| **Sensitivity** | 0.99 (0.95 - 1.00) | 0.98 (0.89 - 1.00) | 0.96 (0.87 - 0.99) | 0.93 (0.83 - 0.97) |
| **Specificity** | 0.09 (0.04 - 0.19) | 0.16 (0.07 - 0.33) | 0.25 (0.13 - 0.44) | 0.34 (0.19 - 0.53) |
| **Positive predictive value** | 0.08 (0.08 - 0.09) | 0.09 (0.08 - 0.10) | 0.10 (0.08 - 0.11) | 0.10 (0.08 - 0.13) |
| **Negative predictive value** | 0.99 (0.96 - 1.00) | 0.99 (0.96 - 1.00) | 0.99 (0.96 - 1.00) | 0.98 (0.96 - 0.99) |
| **Positive likelihood ratio** | 1.09 (1.00 - 1.18) | 1.18 (1.01 - 1.34) | 1.29 (1.05 - 1.54) | 1.43 (1.10 - 1.75) |
| **Negative likelihood ratio** | 0.14 (0.03 - 0.43) | 0.15 (0.04 - 0.41) | 0.17 (0.05 -0.44) | 0.22 (0.09 - 0.44) |
| **Proportion of patients spared intensive testing** | 8 (4 - 18) | 15 (7 - 31) | 24 (12 - 41) | 32 (18 - 50) |

The model results in the presented estimates when a post-salt loading test aldosterone cut-off value of ≥190 pmol/L is applied. The positive likelihood ratio tells you how much to increase the probability of having a disease, given a positive test result. The negative likelihood ratio tells you how much to decrease the probability of having a disease, given a negative test result. The proportion of patients spared intensive testing is the proportion of patients with a predicted probability equal to or below the cut-off value. Estimates and Bootstrap-based 95% confidence intervals are presented for different cut-off values of the predicted probability by the diagnostic tool.
