# Supplementary figures and images for: Development of a clinical decision tool to reduce diagnostic testing for primary aldosteronism in patients with difficult-to-control hypertension
Source: BMC Endocr Disord. 2020 Apr 29;20:56. doi: 10.1186/s12902-020-0528-3 (PMC7191700; doi:10.1186/s12902-020-0528-3)

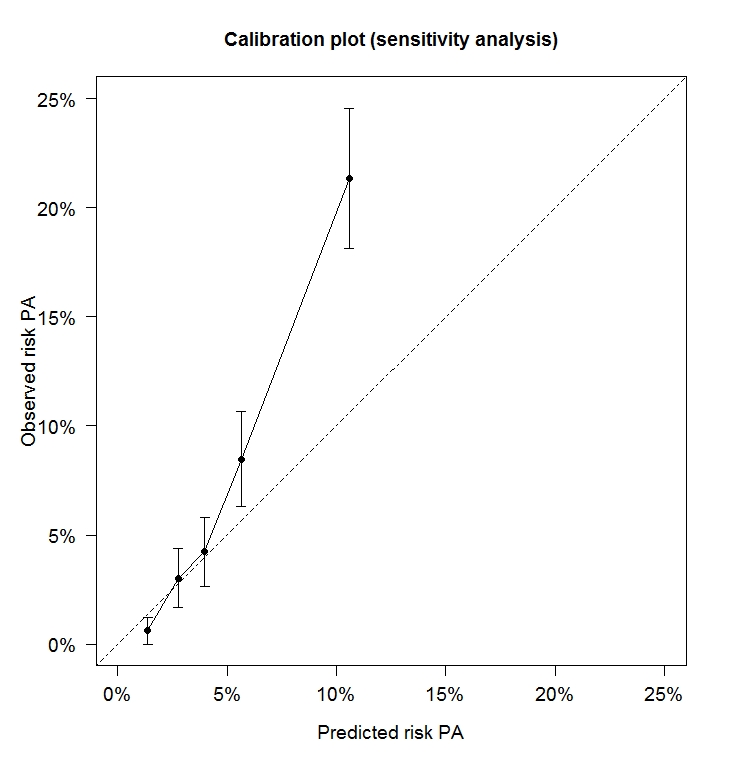

Supplement: Supplementary file 4 — Additional file 4. Calibration plot showing the agreement after sensitivity analysis. It shows the agreement between predicted and observed probabilities of primary aldosteronism when a post-salt loading test aldosterone cut-off value of ≥190 pmol/L is applied. The error bars represent the corresponding Bootstrap-based standard errors. PA = primary aldosteronism. [file 12902_2020_528_MOESM4_ESM.jpeg]
